# A case of severe subcutaneous emphysema in the post-operative period following cleft lip surgery

**DOI:** 10.4103/0019-5049.63638

**Published:** 2010

**Authors:** B Vijayakumar, R Ganessan, V Anbalagan

**Affiliations:** K.A.P.V. Government Medical College and Hospital, Tiruchirapalli, TN, India

**Keywords:** Airway compromise, subcutaneous emphysema, pneumomediastinum, post-operative nausea vomiting

## Abstract

Subcutaneous emphysema is not an unknown complication following cleft lip surgery. We describe a case of severe subcutaneous emphysema that developed six hours after surgery. The laryngoscopic intubation was smooth. Following subcutaneous emphysema the patient was treated conservatively with mask oxygen and spontaneous resolution occurred within 48 hours.

## INTRODUCTION

Subcutaneous emphysema is a rare complication following lip surgery. General anaesthesia may induce nausea and vomiting in the immediate post-operative period. Subcutaneous emphysema following nausea and vomiting is also a rare complication,[1] but it may lead to airway compromise[2,3] adding risk to the life of the patient. Here we describe a case of subcutaneous emphysema following cleft lip surgery, which was induced by retching and vomiting.

## CASE REPORT

A seven-year-old boy, weighing 15 kg, who had both cleft lip and cleft palate, was posted for correction of cleft lip and anterior palate. Pre-operative routine investigations were within normal limits. The patient's airway examination showed Mallampati class 1 (with bifid soft palate). Clinically, the cardiovascular and respiratory systems were normal. The patient was premedicated orally with Tab. Midazolam 2.5 mg and Tab. Ranitidine 50 mg, three hours prior to surgery. The intravenous line was secured. The monitors connected before induction included — 3-lead ECG, pulse oximeter and non-invasive BP. The patient was induced with an intravenous injection of propofol 30 mg along with injection atropine 0.3 mg, followed by injection fentanyl 30 mcg. Injection vecuronium 2 mg was used for intubation. The patient was intubated with a 5 mm size cuffed oral flexometallic ET tube with the help of a size 3 McIntosh blade. The intubation was smooth and atraumatic. Bilateral air entry was confirmed. The tube was fixed in the middle of the lower lip. Anaesthesia was maintained with 0.5 to 2 % halothane, 66%N_2_O and 33% O_2_, via Jackson Ree's modified Ayre's T piece. The procedure lasted for two hours. The vitals were maintained throughout the procedure. The neuromuscular blockade was reversed with intravenous injection neostigmine 0.75 mg and atropine 0.3 mg. The patient was extubated after thorough suctioning, as the patient showed adequate clinical signs of recovery. The patient was kept in the recovery room under close observation.

After 15 minutes the patient developed severe retching for which injection IV metoclopromide 3 mg was given. In spite of that the patient continued to have severe retching, and vomited six times within a period of the next six hours and the vomitus had a bloody tinge. Six hours later, the child developed swelling in the neck and face which extended to the upper thorax. There was extensive crepitus over the area of the swelling. The child was not dyspnoeic, but complained of left-sided chest pain. The respiratory rate was 24 per minute and saturation was 95 – 98 % on room air. The trachea was in the midline and bilateral air entry was present on auscultation.

A chest X ray was taken, which showed subcutaneous emphysema, but there was no evidence of pneumothorax or pneumomediastinum. Oxygen was administered via nasal cannula at 4 l/min and the child was kept under close observation. Injection paracetamol 150 mg was given for pain relief.

Computed tomography (CT) of the neck and upper thorax taken on the next day showed minimal pneumomediastinum and subcutaneous emphysema, but no pneumothorax. Spontaneous resolution of the subcutaneous emphysema occurred in 48 hours.

Oesophagography with barium contrast was performed on the fourth day, which showed no evidence of oesophageal rupture. Bronchoscopy was not done, as the parents were not willing for further investigations and the child was discharged on the seventh day.[Figure 1a and b]

**Figure 1a:**
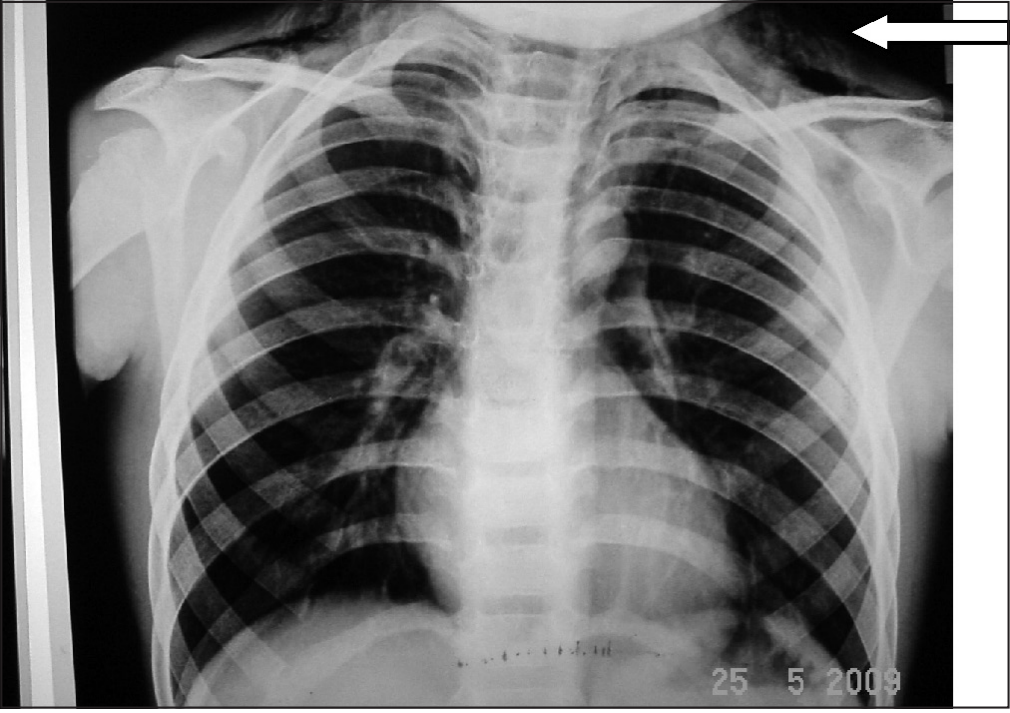
X-ray picture of the patient showing subcutaneous emphysema (marked by arrow) but no pneumothorax

**Figure 1b:**
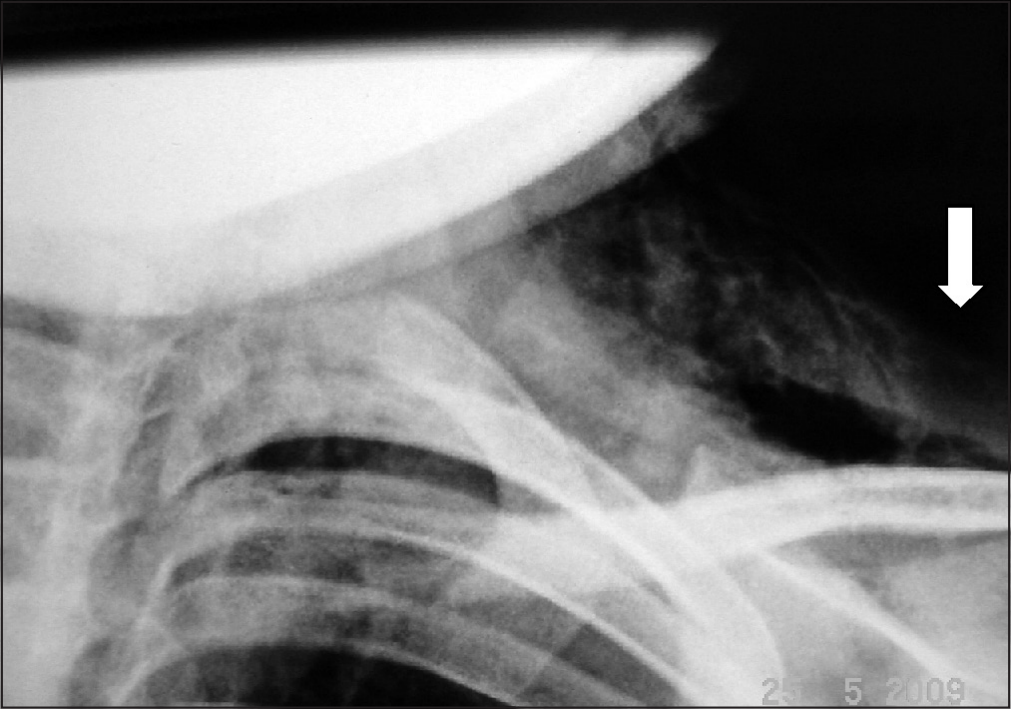
A closer view of the X-ray picture showing subcutaneous emphysema (white arrow)

## DISCUSSION

Differential diagnoses for subcutaneous emphysema following vomiting are spontaneous rupture[4‐7] of the oesophagus caused by vomiting (Boerhaave's syndrome), mediastinitis and a triad of vomiting, chest pain and subcutaneous emphysema,[2,3] trauma to the trachea or hypopharynx and alveolar rupture. In our patient there was chest pain, but the oesophagogram was normal. Figure 2a and b, shows subcutaneous emphysema & pneumo mediastinum in the child.

**Figure 2a:**
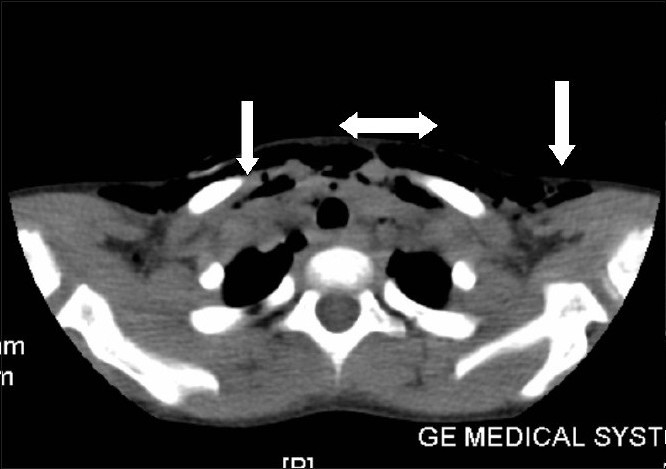
CT picture upper thorax showing subcutaneous emphysema (marked in this picture with white arrows)

**Figure 2b:**
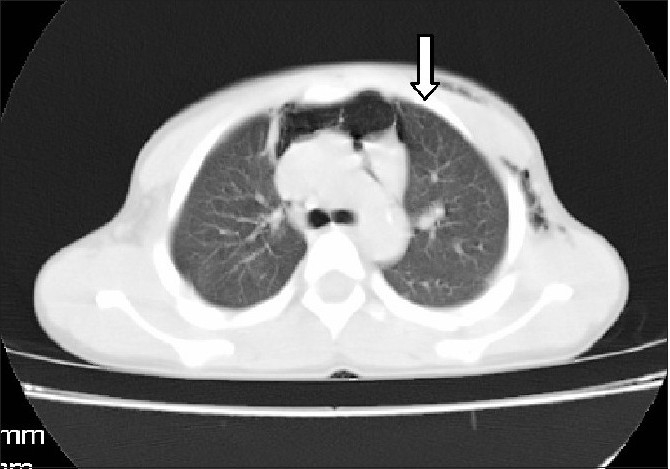
CT picture showing pneumomediastinum (white arrow)

Trauma to the soft tissues of the pharynx, hypopharynx and trachea can occur[8,9] during traumatic intubation, over inflation of the ET tube cuff, repeated intubation attempts, re-positioning of the ET tube without deflating the cuff, stylet protruding beyond the ET tube traumatizing the trachea, patient movement or coughing during intubation and patient head and neck movement after intubation.

Patient factors in addition to tracheal injury[10,11] can be congenital tracheal anomalies, weakness of the membranous trachea, chronic use of steroids and chronic obstructive pulmonary disease. None of the above-mentioned factors apply to our patient and situation. Moreover, the CT shows no evidence of tracheal injury.

Therefore, a possible sequence of events could have been Valsalva's manoeuver during retching and vomiting causing an increase in alveolar pressure that could have led to alveolar rupture. Air could have entered the interstitial spaces and travelled up along the pulmonary vascular sheaths to enter the mediastinum. It could have dissected the soft tissue planes of the neck, to produce a pneumomediastinum and subcutaneous emphysema.[12,13] Classically emphysema starts in the lower part of the chest in an alveolar rupture and progresses towards the neck region.

In this case the CT showed a strong evidence of pneumomediastinum, but no pneumothorax, lung pathology, or pleural effusion. However, pneumomediastinum could also result from air escaping from the injured upper respiratory tract,[14,15] intrathoracic airways,[16] or gastrointestinal tract.[17‐19] Most probably, in this case, it could have been due to microscopic alveolar rupture. Chest pain could be explained by stretching of the mediastinal tissues, which could get aggravated by movement and position change.[18,19] The haemodynamic stability was due to the absence of pneumopericardium. Pneumomediastinum could be confirmed with the help of a chest radiograph; however, the sensitivity was low. The patient recovered completely within 48 hours with conservative treatment, as there was no pneumothorax or pneumopericardium.

To conclude, the exact source of pneumomediastinum and subcutaneous emphysema could not be established in spite of investigations in this case. A normal oesophagogram and atraumatic intubation would make microscopic alveolar rupture a probable source of air. However, the root cause of all the events most probably was retching and vomiting. Therefore attempts should also be made to prevent this, either with the use of pharmacological agents like ondansetron or physically prevent blood leaking into the stomach or avoiding distention of the stomach with ventilated gases or by limiting the use of opioids.
